# Comparative effectiveness of epilepsy surgery versus additional anti-seizure medications for Lennox–Gastaut syndrome: study protocol for a multicenter, mixed-methods study

**DOI:** 10.3389/fneur.2025.1569551

**Published:** 2025-06-18

**Authors:** Sandi Lam, Marc Rosenman, Tracy Dixon-Salazar, Kelly G. Knupp, Liu Lin Thio, Taylor J. Abel, William P. Welch, Laurel Reed, Stephanie C. Randle, Rebecca Garcia-Sosa, Jason S. Hauptman, Carolyn C. Foster, Elizabeth R. Alpern, Lu Zhang, Nicole Villalba, Maura Carroll, Anup D. Patel

**Affiliations:** ^1^Division of Pediatric Neurosurgery, Department of Surgery, Ann & Robert H. Lurie Children’s Hospital of Chicago, Chicago, IL, United States; ^2^Department of Neurological Surgery, Northwestern University Feinberg School of Medicine, Chicago, IL, United States; ^3^Mary Ann & J. Milburn Smith Child Health Outcomes, Research, and Evaluation Center (SCHORE), Ann & Robert H. Lurie Children’s Hospital of Chicago, Chicago, IL, United States; ^4^Department of Pediatrics, Northwestern University Feinberg School of Medicine, Chicago, IL, United States; ^5^Lennox-Gastaut Syndrome (LGS) Foundation, San Diego, CA, United States; ^6^Department of Pediatrics, Division of Neurology, University of Colorado School of Medicine Anschutz Medical Campus, Aurora, CO, United States; ^7^Department of Neurology, Washington University School of Medicine, St. Louis, MO, United States; ^8^Department of Neurological Surgery, University of Pittsburgh School of Medicine, Pittsburgh, PA, United States; ^9^Division of Pediatric Neurosurgery, University of Pittsburgh Medical Center Children’s Hospital of Pittsburgh, Pittsburgh, PA, United States; ^10^Division of Neurology and Child Development Unit, Department of Pediatrics, University of Pittsburgh School of Medicine, Pittsburgh, PA, United States; ^11^Division of Child Neurology, University of Pittsburgh Medical Center Children’s Hospital of Pittsburgh, Pittsburgh, PA, United States; ^12^Division of Pediatric Neurology, Department of Pediatrics, Michigan Medicine, University of Michigan, Ann Arbor, MI, United States; ^13^Pediatric Neurology Department, C.S. Mott Children’s Hospital, Ann Arbor, MI, United States; ^14^Division of Pediatric Neurology, Department of Neurology, University of Washington, Seattle, WA, United States; ^15^Division of Pediatric Neurology and Epilepsy, Department of Pediatrics, Seattle Children’s Hospital, Seattle, WA, United States; ^16^Division of Neurology, Department of Pediatrics, Northwestern University Feinberg School of Medicine, Chicago, IL, United States; ^17^Division of Pediatric Neurology, Ann & Robert H. Lurie Children’s Hospital of Chicago, Chicago, IL, United States; ^18^Division of Neurological Surgery, Phoenix Children’s Hospital, Phoenix, AZ, United States; ^19^Division of Pediatric Emergency Medicine, Ann & Robert H. Lurie Children’s Hospital of Chicago, Chicago, IL, United States; ^20^Division of Neurology, Nationwide Children’s Hospital, Department of Pediatrics, The Ohio State University College of Medicine, Columbus, OH, United States

**Keywords:** Lennox–Gastaut syndrome, epilepsy, epilepsy surgery, anti-seizure medications, comparative effectiveness study

## Abstract

**Introduction:**

Lennox–Gastaut Syndrome (LGS) is a severe developmental epileptic encephalopathy without a known cure. Management of symptoms requires substantial care. Treatment options include anti-seizure medications, dietary therapy, and epilepsy surgery. Two main treatment pathways for patients with LGS with drug resistant epilepsy are additional anti-seizure medications or epilepsy surgery, which have been reported to be effective in reduction of seizure burden and improving quality of life. No studies have directly compared the outcomes of using epilepsy surgery versus using additional anti-seizure medications for the treatment of LGS.

**Methods:**

This study is a multicenter, mixed-methods comparative effectiveness study of LGS patients who have undergone epilepsy surgery or have received an LGS-approved medication for treatment resistant seizures. Aim 1 will analyze the effect of surgical therapies and additional medication on two clinical outcomes: (1a) seizure-related healthcare utilization, and (1b) expressive communication, behavior, and parent-reported quality of life. Based on electronic health record review and coding validation as part of Aim 1a, we will develop computable phenotypes for LGS. The phenotypes will inform the analyses in Aim 1a and Aim 2. Aim 2 will describe the real-world utilization of these treatments across multiple healthcare institutions in the United States. Data will be collected from electronic health records, data marts in the National Patient-Centered Clinical Research Network (PCORnet®) format, caregiver surveys, and focus groups.

**Discussion:**

This study of LGS will provide currently unavailable evidence concerning the real-world comparative effectiveness of epilepsy surgeries and additional anti-seizure medications. The outcomes are those that families identify as important: emergency medical care for seizures and patients’ functional outcomes. The results of this study may help guide decisions regarding the treatment of LGS and development of computable phenotypes for this rare disease. This study using PCORnet® data will also lay the groundwork for future large-scale studies on LGS and other rare epilepsies.

**Clinical trial registration:**

ClinicalTrials.gov, identifier NCT05374824.

## 1 Introduction

Lennox–Gastaut Syndrome (LGS) is a severe developmental epileptic encephalopathy that affects an estimated 48,000 children and adults in the United States (US) (1). LGS is characterized by multiple seizure types, life-long treatment resistant seizures, developmental delay, cognitive and behavioral impairments, and early death (2–4). The onset of seizures is typically in the first 2 years of life, but the syndrome is often not fully recognizable until children are 3 to 5 years old (2, 5). Individuals with LGS have multiple types of seizures including tonic–clonic, tonic, and atonic seizures (2). Prolonged seizures are common and require the use of rescue medications, emergency department (ED) visits, and unplanned hospital admissions (6, 7).

LGS, although rare, accounts for a disproportionate amount of healthcare utilization and expenditures. It accounts for up to 10% of all childhood-onset epilepsies and 1–2% of all epilepsies; however, it represents one-fifth or more of treatment resistant pediatric epilepsy (2, 8–10). Among children and adults, expenditures are two or more times greater in association with LGS than with other forms of epilepsy (11), and all-cause mortality in young people with LGS is ten times that in the general population (3). In a survey about living with LGS, 75% of parents reported that their child experienced seizures every day in the prior week, 30% reported going to an ED at least once in the past 6 months, and 51% reported using rescue medication to stop a seizure in the past 6 months (7). In addition to the burdens created by seizures, people with LGS have a profound level of cognitive and physical impairment. A caregiver-driven survey of young people with severe epilepsies found that in the subgroup with LGS, 42% did not walk independently, 25% did not have functional hand grasp, 42% were entirely dependent on someone else for feeding, 55% did not effectively communicate, and 50% were nonverbal (4). This degree of impairment further increases medical burden and financial stress with hidden costs of care, and significantly impacts the quality of life of patients with LGS and their families (12, 13).

Currently, no cure for LGS exists, and available treatments for LGS are directed at symptom management (6, 14). Treatment options include anti-seizure medications, dietary therapy, and epilepsy surgery. The two primary therapy pathways are surgical approaches, which include Vagus nerve stimulation (VNS), other forms of neurostimulation, and corpus callosotomy (CC), and anti-seizure medications approved for the treatment of LGS. There is a significant gap in current evidence regarding the recommended treatment journey for patients with LGS who experience many seizures each week and have not responded to multiple medications. Each therapeutic approach has been found to have a moderate impact on seizure frequency (15–19). Apart from a small, nonrandomized retrospective study of VNS versus CC (20), none of the approaches have been directly compared to determine which is more effective. Currently, decisions about which treatment strategies to employ rely on studies with significant heterogeneity in their study populations and in their outcome measures (17, 18). In the case of surgery, evidence rests mostly on uncontrolled retrospective and prospective series showing before-and-after comparisons and partly on clinical trials with limited representativeness and generalizability (15, 20–24). Few studies have investigated whether epilepsy surgery or another medication will improve a child’s cognitive and functional profile. A study of 16 patients with “LGS-like” epilepsy suggested improvements in cognition and behavior 6 months after Vagus nerve stimulator implantation (25).

Parents making treatment decisions for a child with LGS face frightening and potentially dangerous options. Baca et al. highlighted the emotional and informational hurdles parents must overcome before deciding to pursue surgery (26). The decision to add medication or to undergo surgery is not simple. Parents and providers question whether surgery – with its associated preparations, stress, intraoperative risks, and risks of post-surgical complications – outweighs the benefits of adding medication. Adding medication is also not simple, because these medications are associated with significant, even dangerous adverse effects individually and in combination. At least one of the medications specifically approved for LGS, felbamate, received a black box warning shortly after its approval in 1993 because of the risk of severe aplastic anemia and hepatic failure (27). All six of the other FDA-approved medications for LGS (clobazam, lamotrigine, topiramate, rufinamide, fenfluramine, and cannabidiol) can have significant adverse effects (18).

Delaying effective treatment may expose the child to longer periods of uncontrolled seizures, leading to greater decline and long-term disability. Early intervention may limit the impact of seizures in the young brain and may result in less impairment later in life (28–34). A comparative effectiveness study is needed to support informed decisions in the management of this challenging disease. A study comparing surgical procedures versus the addition of LGS-approved medication may help guide future decision-making regarding the treatment journey of LGS and may improve patient outcomes.

## 2 Methods and analysis

### 2.1 Study aims

Aim 1: Determine the effect of epilepsy surgery and LGS-approved anti-seizure medications on two key clinical outcomes in children with LGS.

a. Compare the effectiveness of the addition of surgeries versus the addition of another medication for reducing seizure-related emergency healthcare utilization.

b. Compare the effectiveness of these two pathways and of early versus later use of surgery versus medications on measures of children’s expressive communication, behavior, and quality of life.

Aim 2: For individuals with LGS, describe real-world utilization of these treatments across multiple centers in the US, and identify variability including disparities that may currently pose challenges and barriers to ensuring optimum access to and use of appropriate therapies.

### 2.2 Study design

**Figure 1:**
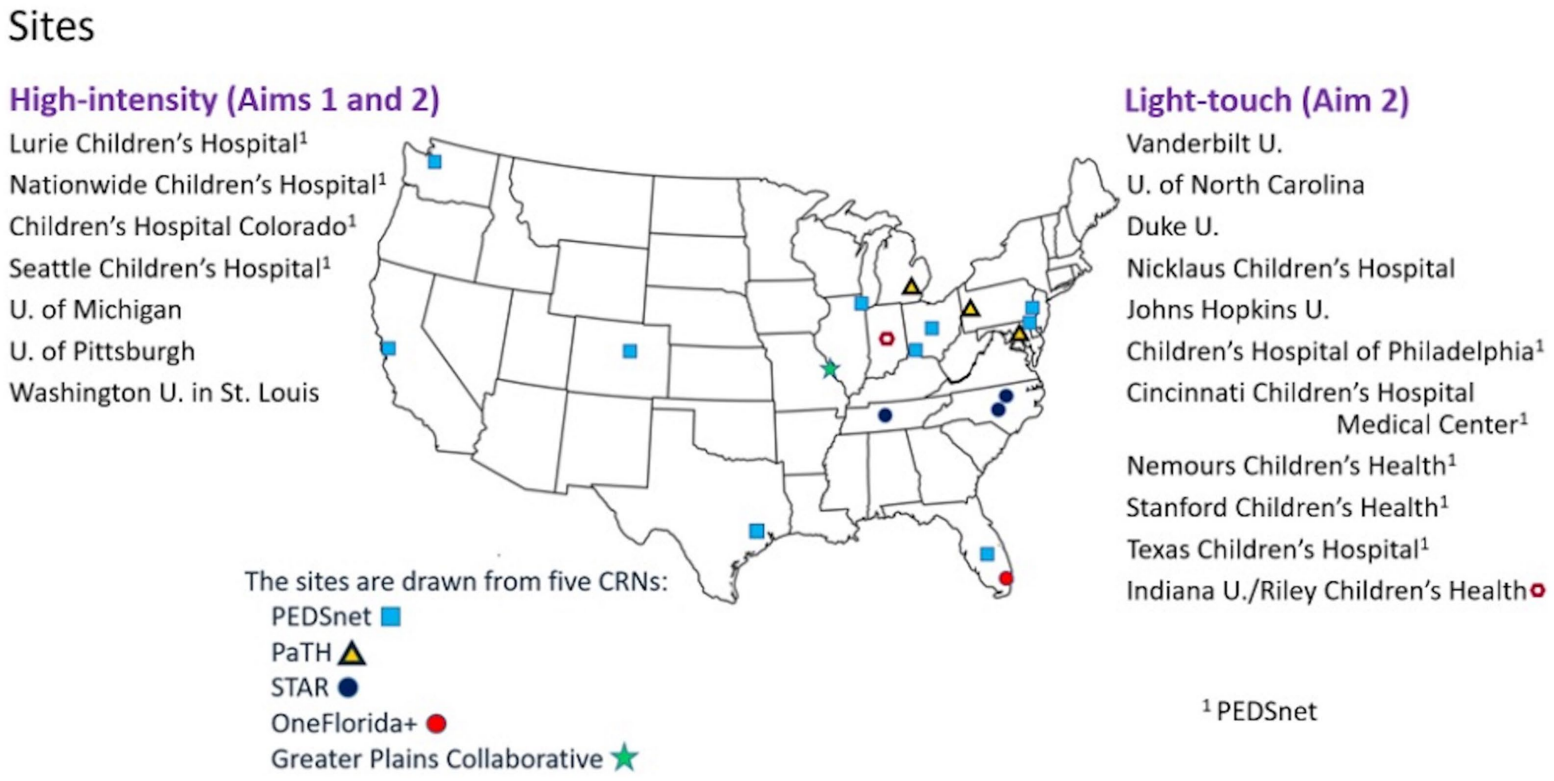
Map of sites. CRNs, clinical research networks; U, University.

This multicenter, mixed-methods comparative effectiveness study will use the Patient-Centered Clinical Research Network (PCORnet®) data mart infrastructure to work with 18 academic pediatric sites across 5 clinical research networks: PEDSnet, a national pediatric learning health system; PaTH Towards a Learning Health System (PaTH); Stakeholders, Technology, and Research (STAR); OneFlorida+; and Greater Plains Collaborative. PCORnet® is a national research network of health systems with access to electronic health records (EHR) data that have been standardized using a common data model (35). The 18 PCORnet® sites we selected have large clinical programs in pediatric epilepsy and serve large numbers of patients with LGS. At 7 high-intensity sites, clinical research teams will conduct EHR review for coding validation of the diagnosis of LGS and will recruit caregivers to complete surveys measuring communication, behavior, and quality of life. Computable phenotypes for LGS will be developed based on elements available in the EHR. Data from an additional 11 sites (light-touch sites) will be pulled and analyzed based on cohort identification by these computable phenotypes (Figure 1).

The comparative effectiveness analyses will be conducted in populations selected in two ways: (1) patients with chart reviewed validated LGS diagnoses, and (2) patients with LGS based on computable phenotypes.

#### 2.2.1 Aim 1a

Through a retrospective chart review study across the 7 high-intensity sites, we will validate the International Classification of Diseases, Tenth Revision (ICD-10) codes for LGS and will develop computable phenotypes for LGS.

In a retrospective cohort study across the 7 high-intensity sites, we will compare the effectiveness of epilepsy surgery versus additional medication for decreasing seizure-related ED visits and inpatient admissions in patients with validated diagnoses of LGS. This will entail a comparison of any surgery versus the medication arm, as well as comparisons of each of the two most common surgical approaches (VNS and CC) to each other and each of those to the medication arm.

#### 2.2.2 Aim 1b

In a cross-sectional study across the 7 high-intensity sites, we will evaluate the two treatment pathways regarding expressive communication, behavior, and parent-reported quality of life of patients with validated diagnoses of LGS, and the impact of earlier (younger age) versus later (older age) utilization of the therapy. Caregivers will complete a series of surveys to assess their child’s communication, behavior, and quality of life.

#### 2.2.3 Aim 2

In a retrospective cohort study across all 18 sites, we will analyze patterns of utilization of surgeries versus additional medication. We will also assess variation in their use across sites by age, race, ethnicity, health insurance, and overall medical complexity. Aim 2 will use the computable phenotypes for LGS that are developed from the intensive coding validation with EHR review and clinical diagnosis adjudication in the retrospective study cohort in Aim 1a.

Institutional Review Board (IRB) review for this protocol will be conducted at each study site, or in some cases via reliance upon a PEDSnet IRB protocol. All aspects of the study (Aims 1a, 1b, and 2) have received approval from the IRB of Ann & Robert H. Lurie Children’s Hospital. This study is registered at www.ClinicalTrials.gov (NCT05374824).

### 2.3 Inclusion and exclusion criteria

Although children in many research studies are defined as <18 or <21 years of age, in practice, children with severe epilepsies such as LGS often remain with their pediatric providers well into their third decade of life, because of severe disability, and may continue to have coverage up to age 26 through their parents’ health insurance plans. Patients in the cohort will have been less-than-or-equal-to 26 years old at the beginning of the study period.

Inclusion and exclusion criteria for Aims 1a, 1b, and 2 are shown in Table 1.

**Table 1:** Study inclusion and exclusion criteria.

Aims	Inclusion criteria	Exclusion criteria
Aim 1a	Patient has a validated diagnosis of LGS or meets one of the LGS computable phenotype definitions the study evaluated Index treatment occurred between 2016 and 2021: first epilepsy surgery or initiation of additional LGS-approved medication Patient received neurological care at a study site at least 1 year before intervention and 2 years after intervention at the site	Patient does not meet minimum follow up criteria for neurological care at the study site
Aim 1b	Patient has a validated diagnosis of LGS Patient is currently being treated at a study site Caregiver can participate in English or Spanish	
Aim 2	Computable phenotype for LGS based on Aim 1a	

LGS, Lennox–Gastaut syndrome.

### 2.4 Data collection

#### 2.4.1 Aim 1a

**Figure 2:**
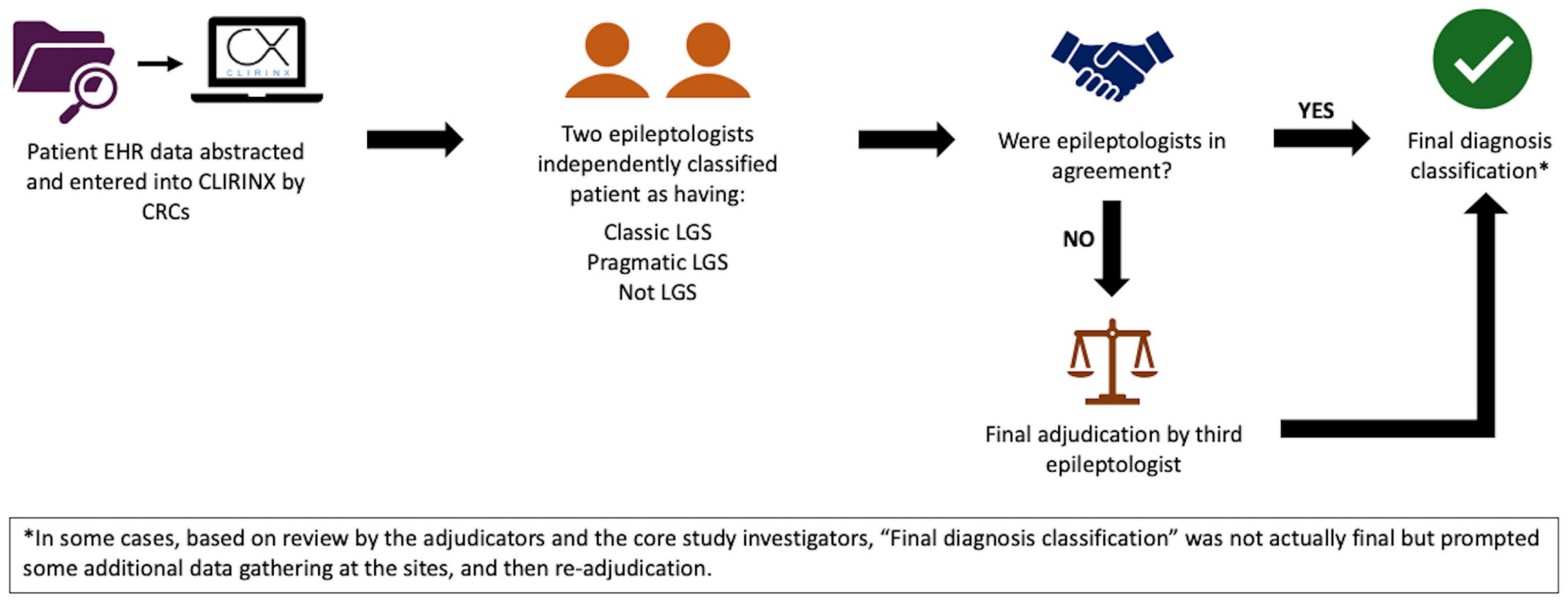
LGS classification workflow in Aim 1a. CRCs, clinical research coordinators; EHR, electronic health record; LGS, Lennox–Gastaut syndrome.

Data for the validation and computable phenotype study will be collected through retrospective chart review. Trained clinical research coordinators or physicians will review the EHR of patients who had an ICD-10 code for LGS (G40.81x) between January 1, 2016, and December 31, 2022, (as well as a sample without G40.81x, as detailed below) at their respective sites and will record key information reflecting diagnostic criteria for LGS into CLIRINX^1^, a secure web-based clinical research informatics platform designed to support data collection for epilepsy and rare disease clinical research. This information includes seizure history, treatment history (and current medications), etiology/antecedent factors, EEG findings, developmental status/intellectual disability, functional abilities, and neurosurgical interventions. Once data are entered for a given patient in CLIRINX, two pediatric epileptologists, from principal sites different than that of the patient, will independently review the data to classify the patient as having (1) classic LGS, (2) pragmatic LGS, or (3) not LGS. Definitions for each classification are shown in Table 2. If discordant, the file will be automatically sent to a third reviewer (ADP) for adjudication. The LGS classification workflow is shown in Figure 2. A sample of patients without an ICD-10 code for LGS but with a nonspecific ICD-10 code often associated with intractable epilepsy (G40.21^2^, G40.41^3^, G40.80^4^, G40.91^5^) will also be reviewed using the same process for data extraction and central review. New computable phenotypes for LGS will be developed using ICD-10 codes, medication history, and other information available in the EHR. Of note, the International League Against Epilepsy (ILAE) definition published in 2022 came after the development of this study protocol and initiation of this study (36). The retrospective study period is 2016 through 2022, the most recent complete year of data at the time this study was initiated.

**Table 2:** LGS classifications in Aim 1a.

Classification	Definition
Classic LGS	Meets classic criteria for LGS: Tonic seizures At least one other generalized seizure type Onset of seizures in the first 5 years of life Generalized slow spike-and-wave on EEG (<3 Hz) Cognitive impairment
Pragmatic LGS	Meets pragmatic criteria, as used in randomized trials, for LGS: At least 2 types of generalized seizures including drop seizures; drop seizures include atonic or tonic–clonic Slow spike-and-wave on EEG (<3 Hz) Cognitive impairment
Not LGS	Does not meet either classic or pragmatic criteria for LGS

EEG, electroencephalography; LGS, Lennox–Gastaut syndrome.

Data on emergency healthcare utilization, as well as treatments and covariates, will be extracted from PCORnet® data marts of the 7 high-intensity sites. The data types include demographics, diagnoses and conditions, encounters, procedures, prescriptions, medication administrations, healthcare providers, and deaths. Seizure-related emergency healthcare utilization will be defined as the frequency per year of seizure-related ED visits and seizure-related inpatient admissions in the 2 years after intervention (surgery or medical), with adjustment for the rate during the year before intervention. A secondary analysis will be done for 1 year after intervention as well. The index date of the intervention will be defined between January 1, 2016, and December 31, 2021, as that of the first relevant surgery or (if there was no surgery) that of the first exposure to a third anti-seizure medication, with at least one of the three being among the seven LGS-approved medications listed above. To the extent possible, we will also control for dietary therapy as a variable collected by chart review and accessible by codes. We also will analyze all emergency healthcare visits and inpatient visits (with or without seizure diagnosis codes) and the subset for which there is a diagnosis of fracture, head trauma, lacerations/avulsions/contusions, burns, aspiration, aspiration pneumonia, or near drowning (37), as an expanded seizure-related definition. Subset analysis will be done on data from 2020 and 2021, the years of the COVID-19 pandemic (38).

#### 2.4.2 Aim 1b

Communication, behavior, and quality of life will be measured using survey instruments determined by community-engagement and stakeholder participation, which will include a series of focus groups of caregivers, advocacy group members, and clinicians. The rationale, methodology, and results of the focus group input will be documented and reported. The goal is for surveys to be available in English and Spanish.

Surveys selected *a priori* to include in the focus group discussions include and are not limited to the following for each domain.

Communication: The Communication Matrix, Communication and Symbolic Behavior Scales.

Behavior: Adaptive Behavior Assessment System, Aberrant Behavior Checklist, Vineland 3.

Quality of Life: Quality of Life Inventory-Disability Questionnaire, CDKL-5 Severity Assessment.

Final selection of the validated survey instruments will be made by synthesizing the input from the focus groups, which will evaluate relevance and importance to families with lived experience, as well as comprehensibility and feasibility of completing the surveys, including cognitive load, validity, and time burden.

#### 2.4.3 Aim 2

Data on epilepsy surgery and anti-seizure medication prescriptions will be extracted from the PCORnet® data marts of all 18 sites from a 7-year period (January 1, 2016, to December 31, 2022). This period will be extended when additional years are available, and the maximum index date will be moved accordingly to 1 year before the end of the data. Epilepsy surgery will include cranial epilepsy surgery, CC, VNS, deep brain stimulation (DBS), and responsive neurostimulation (RNS). Patient data such as center, age, race, ethnicity, health insurance, and medical complexity will also be extracted.

### 2.5 Sample size estimates

Preliminary data exploration across the 7 high-intensity sites identified an average of over 200 pediatric patients with LGS per site. Based on this, we anticipate a total sample size of more than 3,600 with ICD-10 diagnosis code G40.81x and, after sampling, chart review, and recruitment, approximately 464 participants for Aim 1b. We expect that approximately one-third will be in the surgery arm.

### 2.6 Data analysis

#### 2.6.1 Aim 1a

Agreement before and after consensus will be quantified with Cohen’s Kappa. The gold standard diagnosis of LGS will serve as the binary outcome variable, which is inclusive of either classic LGS or pragmatic LGS. We will develop computable phenotype models containing candidate factors available in EHR repository data (such as diagnosis codes, medications, and combinations thereof) that may discriminate between patients with and without LGS. We will compute the sensitivity, specificity, and the area under the curve. We anticipate identifying three computable phenotypes: one that optimizes sensitivity while maintaining acceptable specificity, one that optimizes specificity while maintaining acceptable sensitivity, and one that balances the two. Our goal is that this work would inform and could be applied in the selection of future study cohorts based on EHR or administrative data.

To test the computable phenotypes further, we will perform our primary analysis of ED and inpatient admissions with our gold standard definition of LGS and with each of the computable phenotype definitions. The computable phenotype that provides the least distortion from that measured using the gold standard may be used as the primary computable phenotype; however, we anticipate replicating all analyses with all computable phenotypes to account for the possibility that unanticipated multivariable interaction, or other issues perhaps related to data quality or completeness, may affect the results.

The primary analyses will compare epilepsy surgery (CC or VNS, CC alone, VNS alone) to the addition of another LGS-approved medication. We also will compare CC to VNS. While CC and VNS are the most common surgeries, we will also explore all epilepsy surgeries including cranial epilepsy surgery, CC, VNS, DBS, and RNS to addition of another LGS-approved medication, to understand VNS and CC in the spectrum of care. We will use a generalized linear mixed model with Poisson distribution and log link to compare the frequency of ED visits and inpatient admissions in the 6 months post-intervention. To guard against any potential biases in the selection of the type of surgical therapy or of surgery versus medication that might arise due to medical complexity, we will employ inverse probability of treatment weighting (IPTW), which uses the propensity score/conditional probability to balance baseline patient characteristics across different treatment groups. Treatment selection will be modeled with a logistic regression using information available at the time the decision is made like age, sex, pediatric complex chronic conditions (PCCC) (39), and anti-seizure medication use. The inverse of the resulting probability will be used to weight each individual in regression models for the outcomes. PCCC scores, reflecting medical complexity, will be calculated from existing data provided by each site. Based on our knowledge of and experience with LGS and its treatment, we do not expect large differences between the patients receiving the different types of therapies. We will describe what the differences are that appear to influence the selective use of these different therapies.

A random site effect will account for clustering within sites. Robust standard errors will be used to control for mild violation of distributional assumptions. In the case of severe violations, a Negative Binomial or zero-inflated Poisson model may also be considered. We will report the conditional rate ratios that estimate the expected rates of ED visits and separate hospitalizations in the surgery arm compared to the medication arm for the 1-year and 2-year periods following intervention. Additionally, a combined variable representing overall emergency healthcare utilization, including both ED visits and inpatient admissions, will be considered.

#### 2.6.2 Aim 1b

The primary analysis will employ a linear mixed model with surgery versus medication as the independent variable to compare the effect of treatment on communication, behavior, and quality of life. A similar analytic approach will be used for secondary analyses comparing early versus later use of treatment. We will select nonsurgical comparison patients to be matched to surgical patients on a variety of factors that are associated with the choice of therapy. Should we be unable to match certain factors, we will consider separate multivariable adjustments. We will use generalized linear mixed models, with appropriate link and distributional assumptions, and will allow for the separation of within-site and between-site variance components. Results will be reported with and without adjustment for potential confounders. If our final sample size is not adequate for such statistics, we at minimum will provide a baseline descriptive analysis of the results.

#### 2.6.3 Aim 2

We will describe patterns of use and will conduct comparative effectiveness analyses of epilepsy surgery and LGS-approved medications across the multiple sites. In parallel with analyses in Aim 1a, we will consider surgical versus medication pathways as well as CC and VNS separately. We will also explore all epilepsy surgery including cranial epilepsy surgery, CC, VNS, DBS, and RNS to understand the spectrum of real-world care. Results will be summarized overall and by factors of interest (e.g., center, age, race, ethnicity, health insurance, medical complexity). Health insurance will be categorized as public, private, or self-pay. Exploratory analyses will be conducted for comparisons. For categorical variables, proportions will be reported, and Chi-square tests or Fisher’s exact tests will be used to assess independence. For continuous variables, means and standard deviations will be reported, and t-tests will be performed to evaluate independence. These data will provide context for interpreting the generalizability of the analyses performed for Aims 1a and 1b.

## 3 Discussion

While various treatment options exist for LGS, there have not been comparative effectiveness studies regarding epilepsy surgery versus additional medications for LGS patients who do not respond to initial treatments. Clinical guidelines for LGS focus on medications (14, 40–42). Treatment decisions are often guided by judgment based on a clinician’s own experience (6, 43). Decisions are also made by comparing the results of drug trials conducted in different eras, in different settings, using different selection criteria, and sometimes with different definitions of LGS (17, 18). For epilepsy surgery, the level of evidence is weak, including uncontrolled retrospective and prospective studies showing before-and-after comparisons (15, 20–24). Our study will fill this gap by providing head-to-head comparisons among the potential therapies used for LGS (44, 45).

### 3.1 Computable phenotype

Validation of ICD-10 coding for LGS based on review of the EHR and clinician adjudication has not been previously published. It can be challenging to accurately diagnose LGS. The clinical presentation of the syndrome evolves, and the definition of LGS has changed over time (6, 44–47). There is a wide range of presentations, and the time period before the characteristic features of LGS manifest varies between individuals (6, 8, 44–46, 48). Many of the features of LGS overlap with those of other severe epilepsies, which can lead to misdiagnosis or delayed diagnosis (46), and can affect treatment decisions and outcomes. The definition of LGS in trials has also varied, which makes it difficult to fully interpret the effectiveness of treatments (17, 18, 43). We aim to develop methods to classify patients consistently and accurately within large datasets. Such phenotypes would facilitate future large-scale LGS studies that may lead to new insights into the treatment of LGS. They may also enable more accurate diagnosis of LGS in the clinical setting.

### 3.2 Community-engaged research

Engaging patients and their families in research is an important component of our study. The patient and family journey is a critical motivating factor and is indeed the basis of this project. Engagement enhances the overall quality of research by improving the relevance and applicability of study outcomes (49). A Stakeholder Advisory Board (SAB) consisting of clinicians, caregivers, patients, and patient advocates is working together with the investigator team to help generate research questions, assist in the conduct of research, monitor progress, and help disseminate information. The primary purpose of the SAB is to ensure that the study design and research activities are patient-centered and informed by and relevant to patients and families with lived experience. As an example, a series of focus groups will be conducted together to select the survey instruments for outcome measurements to be used for Aim 1b.

### 3.3 PCORnet® data

This study will also enhance PCORnet®‘s infrastructure and capability to conduct research on LGS and other rare pediatric epilepsies by developing a robust platform and network for future research. We will establish a multi-site pediatric epilepsy research group that will engage with patients, parents, and community stakeholders. Creating a comprehensive database and collaboration among 18 sites will facilitate large-scale studies that were previously unattainable for these rare conditions. This network will support collection and analysis of high-quality data, promote sharing of resources among researchers, and improve understanding of data provenance and data quality for pediatric epilepsy research in PCORnet®. Through intensive reviews of medical records, we will improve techniques for computable phenotyping of LGS. We seek to harmonize such phenotyping across the leading US centers for pediatric epilepsy.

### 3.4 Limitations

There are limitations. Retrospective studies can have bias due to incomplete or inaccurate historical data. The reliance on self-reported survey data in Aim 1b can lead to response and recall biases. The open cohort design can introduce selection bias due to participants entering at different times. These factors may limit the ability to draw definitive conclusions and may affect the generalizability of our findings. Future research should aim to address these limitations by incorporating prospective data collection. Nevertheless, this is the first national scale comparative effectiveness study addressing the choice of epilepsy surgery or additional anti-seizure medications: the study is designed to address outcomes that matter to patients and families and will inform future patient-centered LGS research including prospective studies.

Previously, this study proposal approved for PCORI sponsorship was entitled, “Comparative effectiveness of palliative surgery versus additional anti-seizure medications for Lennox–Gastaut Syndrome.” There is growing recognition in the field that the terminology of palliative epilepsy surgery needs to be updated: while treatment is not expected to be curative, outcomes of reduced seizure burden and improved quality of life can be worthwhile and life-changing. The historical and common use of the term palliative epilepsy surgery may be misleading for epilepsy care, and we shall be mindful to rectify the terminology in ongoing and future dissemination efforts. To this end, we have submitted a request to revise the study title to “Comparative effectiveness of epilepsy surgery versus additional anti-seizure medications for Lennox–Gastaut Syndrome,” and we have removed the use of the term “palliative” in this manuscript.

The study design is strengthened by validation and adjudication of the diagnosis of LGS. Since our cohort will be representative of a broader community of LGS patients who receive care at sites across the US, our results will be more robust and more generalizable than those in the existing literature. We also will provide the broad community of stakeholders with evidence describing current LGS treatment patterns across 18 centers. This study will strengthen patient/family engagement and epilepsy research collaboration across the US and will help describe variability, disparities, and opportunities to improve care and care coordination.

### 3.5 Ethics and dissemination

The study will be reviewed and approved by the IRB initially at Ann & Robert H. Lurie Children’s Hospital and subsequently at each of the study sites, in some cases via reliance upon a PEDSnet IRB protocol. Participants will provide informed consent to participate in Aim 1b.

Algorithms or methods developed to assist the creation of computable phenotypes for LGS will be transmitted to the Phenotype Knowledgebase website (PheKB, www.phekb.org). All materials developed for activities involving our leadership, patients, and stakeholders will be shared and archived in the Patient-Centered Outcomes Research Institute Engagement Tool and Resources Repository. Copies of programming codes used to derive the data sets and to perform the analyses will also be made available. The data dictionary containing variable names and labels, codes and their meanings, and branching logic will be made available as well. Study resources will also be made available through PCORI-recommended mechanisms. Dissemination of study design, review of interim results, calls for ongoing input, and reports of findings will be done in collaboration with the LGS Foundation and the broader communities.

The PEDSnet data used for this study was provided by PEDSnet and can be shared only with approval from the PEDSnet consortium and an appropriate Data Use Agreement.

## Footnotes

^1^CLIRINX – Clinical Research IT. www.clirinx.com

^2^G40.21 – location-related (focal) (partial) symptomatic epilepsy and epileptic syndromes with complex partial seizures, intractable.

^3^G40.41 – other generalized epilepsy and epileptic syndromes, intractable.

^4^G40.80 – other epilepsy.

^5^G40.91 – epilepsy, unspecified, intractable.
